# Deep neural network to locate and segment brain tumors outperformed the expert technicians who created the training data

**DOI:** 10.1117/1.JMI.7.5.055501

**Published:** 2020-10-16

**Authors:** Joseph Ross Mitchell, Konstantinos Kamnitsas, Kyle W. Singleton, Scott A. Whitmire, Kamala R. Clark-Swanson, Sara Ranjbar, Cassandra R. Rickertsen, Sandra K. Johnston, Kathleen M. Egan, Dana E. Rollison, John Arrington, Karl N. Krecke, Theodore J. Passe, Jared T. Verdoorn, Alex A. Nagelschneider, Carrie M. Carr, John D. Port, Alice Patton, Norbert G. Campeau, Greta B. Liebo, Laurence J. Eckel, Christopher P. Wood, Christopher H. Hunt, Prasanna Vibhute, Kent D. Nelson, Joseph M. Hoxworth, Ameet C. Patel, Brian W. Chong, Jeffrey S. Ross, Jerrold L. Boxerman, Michael A. Vogelbaum, Leland S. Hu, Ben Glocker, Kristin R. Swanson

**Affiliations:** a H. Lee Moffitt Cancer Center and Research Institute, Department of Machine Learning, Tampa, Florida, United States; b Imperial College, Biomedical Image Analysis Group, London, United Kingdom; c Mayo Clinic, Mathematical NeuroOncology Lab, Phoenix, Arizona, United States; d University of Washington, Department of Radiology, Seattle, Washington, United States; e H. Lee Moffitt Cancer Center and Research Institute, Department of Cancer Epidemiology, Tampa, Florida, United States; f H. Lee Moffitt Cancer Center and Research Institute, Department of Diagnostic Imaging and Interventional Radiology, Tampa, Florida, United States; g Mayo Clinic, Department of Radiology, Rochester, Minnesota, United States; h Rhode Island Hospital and Alpert Medical School of Brown University, Department of Diagnostic Imaging, Providence, Rhode Island, United States; i H. Lee Moffitt Cancer Center and Research Institute, Department of Neurosurgery, Tampa, Florida, United States; j Mayo Clinic, Department of Neurosurgery, Phoenix, Arizona, United States

**Keywords:** deep learning, brain tumors, observer studies, segmentation, validation

## Abstract

**Purpose:** Deep learning (DL) algorithms have shown promising results for brain tumor segmentation in MRI. However, validation is required prior to routine clinical use. We report the first randomized and blinded comparison of DL and trained technician segmentations.

**Approach:** We compiled a multi-institutional database of 741 pretreatment MRI exams. Each contained a postcontrast T1-weighted exam, a T2-weighted fluid-attenuated inversion recovery exam, and at least one technician-derived tumor segmentation. The database included 729 unique patients (470 males and 259 females). Of these exams, 641 were used for training the DL system, and 100 were reserved for testing. We developed a platform to enable qualitative, blinded, controlled assessment of lesion segmentations made by technicians and the DL method. On this platform, 20 neuroradiologists performed 400 side-by-side comparisons of segmentations on 100 test cases. They scored each segmentation between 0 (poor) and 10 (perfect). Agreement between segmentations from technicians and the DL method was also evaluated quantitatively using the Dice coefficient, which produces values between 0 (no overlap) and 1 (perfect overlap).

**Results:** The neuroradiologists gave technician and DL segmentations mean scores of 6.97 and 7.31, respectively (p<0.00007). The DL method achieved a mean Dice coefficient of 0.87 on the test cases.

**Conclusions:** This was the first objective comparison of automated and human segmentation using a blinded controlled assessment study. Our DL system learned to outperform its “human teachers” and produced output that was better, on average, than its training data.

## 1 Introduction

Applications of deep learning (DL) in medical imaging have proliferated in the last few years. DL systems have proved particularly effective for segmenting organs and lesions in MRI and CT image volumes. By their nature, DL systems tend to be “black boxes,” unable to provide insight into how their segmentation results were obtained. Consequently, a lingering issue is the reproduction and validation of the many compelling results.

Evaluation of DL-based segmentation has focused primarily on measuring overlap with reference segmentations. Typically, the reference segmentations are created by radiologists or by expert technicians with training in image processing. Often, these segmentations are then reviewed for accuracy by one or more independent radiologists. In essence, this process “front-loads” human expertise to improve the training and assessment of DL systems.

Here, we describe a complementary approach—one that “back-loads” human expertise to evaluate (and potentially improve) the results of DL segmentation. Our system allows comprehensive and objective comparisons of DL and human segmentations via blinded controlled assessment studies. Multiple experts, potentially located across widely separated geographic regions, can easily access our cloud-based system via a common, secure web browser.

We used our system to compare technician-derived and DL-derived segmentations of brain tumors. Often these are heterogeneous, diffuse, and highly infiltrative aggressive tumors. Consequently, it is a time-consuming task to segment brain tumors in MRI scans. Therefore, considerable effort has been devoted over the last 25 years to develop computer-based methods to accelerate and automate brain tumor segmentation.^1^^–^^8^ Recently, effort has focused on designing and training DL systems to segment these complex lesions.^9^^–^^16^ Careful validation of these systems is required to ensure translation to clinical workflows.

This paper includes three primary contributions.

1. It describes the first objective comparison of automated and human segmentation using a blinded controlled assessment study.
2. It provides the first quantitative evidence that an artificial intelligence algorithm can outperform human experts on an image segmentation task.
3. It demonstrates that DL systems can learn to produce lesion segmentations that are more accurate than their training data.

The last finding contradicts the common belief that “a model is only as good as the data used to train it.” In Sec. 4, we postulate why this is often not the case and why this finding may generalize to other organ and lesion segmentation tasks. Finally, we suggest some new strategies for creating medical segmentation training data.

## 2 Materials and Methods

### 2.1 Data

This study was reviewed and approved by the Mayo Clinic Institutional Review Board. Over the last 15 years, we have been collecting and segmenting routine clinical MRI exams of brain tumor patients. This collection supports ongoing research into mathematical modeling of brain tumor growth.^17^ Our brain tumor database contains 70,542 MRI studies (imaging time points) from 2892 unique patients. These studies range in date from 1986 through 2019 and were acquired on both 1.5 and 3 T MRI systems. Our image analysis team, currently 15 technicians, has segmented brain tumors in 38,535 of these time points.

Image analysts undergo a training program to ensure consistent performance. The underlying principle of the training is to learn, internalize, and apply complex rule sets across all MR modalities. Each rule set is based upon selecting the bright signal due to tumor presence as opposed to a bright signal due to normal or abnormal nontumor brain tissues. Each of these segmentations has been reviewed for accuracy by a segmentation supervisor prior to inclusion in the database. The supervisor has extensive experience segmenting brain tumors but is not a board-certified neuroradiologist. However, a neuroradiologist is available for consultation.

For this proof-of-concept experiment, we restricted the analysis to pretreatment MRI studies since treatment may cause significant alterations to brain appearance. That, in turn, may cause ambiguities in the manual segmentations, which could impact our segmentation evaluation study. Our database was searched to identify pretreatment studies that included both a T1 postcontrast (T1c) scan along with a fluid-attenuated inversion recovery (FLAIR) scan. Both the T1c and FLAIR scans also had to have at least one segmented region each. We identified 914 pretreatment MRI studies from our brain tumor database. Of these, 741 met these inclusion criteria.

Some scans had multiple segmentations, each performed by a different technician. When two segmentations were available for a given scan, we used the intersection of the two regions. When more than two segmentations were available, they were combined into a consensus segmentation using majority voting, per voxel. Each tumor was segmented into two compartments: enhancing signal on T1c and bright signal on FLAIR. However, the use of two segmentation compartments greatly increased the cognitive burden during the visual assessment study (described below). Therefore, the two regions were combined into a single whole-tumor region using the union of the two compartments via a logical “OR” operation, per voxel.

### 2.2 Preprocessing

Each included study was processed using the following fully automated pipeline: (1) the MRI volumes and brain tumor segmentation files were copied from the database; (2) the extracted data were verified to ensure completeness; (3) the FLAIR volume was rigidly coregistered to the T1c volume using the SimpleElastix framework;^18^ (4) each volume was resampled to a common voxel spacing of 1×1×2  mm (x,y,z). We compared trilinear and tricubic interpolation for resampling. There was little visible difference between the two methods, likely because the target voxel size was smaller than the source voxel size for the majority of exams. Therefore, we selected trilinear interpolation; (5) contrast-to-noise ratio was improved using nonlinear curvature-flow noise reduction;^19^ (6) radiofrequency nonuniformity was reduced using the N4 algorithm;^20^ (7) the brain was masked within the head (“skull-stripped”). This process is described and compared with other methods in more detail in a recent publication by our team;^21^ (8) the MR intensities of brain voxels were adjusted to have zero mean and unit variance; (9) the T1c and FLAIR segmented regions were combined using a per-voxel logical OR operation to create a binary mask representing the combined tumor region; and (10) the Harvard-Oxford probabilistic atlas^22^ was nonlinearly transformed to fit the subject’s brain.

The atlas was composed of two components: cortical and subcortical regions. We used the 1-mm isotropic voxels, maximum probability version in our experiments. Atlas alignment was accomplished using the SimpleElastix framework, following a procedure described previously.^23^ Briefly, it involves two steps: an initial affine transformation to coarsely align the International Consortium of Brain Mapping 152 (ICBM152) template^24^ to the subject brain, followed by a nonlinear local b-spline transformation to refine the alignment. Since the Harvard-Oxford atlas is itself aligned with the ICBM152 template, the composite transformation used to align the template with the subject’s brain may be used to align the atlas with the subject’s brain. This process is known to have limitations, especially when significant alteration or pathology is present in a subject’s brain.^25^ Consequently, our intent was to use the aligned atlas as an aid for visualization.

### 2.3 Network Architecture and Training

The training set was used to train the open-source 3D “DeepMedic” convolutional neural network, described elsewhere.^9^^,^^26^ This network has achieved state-of-the-art results in the international multimodal BraTS challenges.^10^

Network training is controlled via a large number of configurable parameters. Unless otherwise noted below, we used default parameter values described in detail in the software documentation. These parameters have been pretuned for BraTS. In particular, the training loss was voxel-wise cross entropy as commonly used for segmentation tasks,^26^ the number of subepochs per epoch was fixed at 20, the initial learning rate was fixed at 0.001, and the step decay factor was fixed at 2. Intensity augmentation was performed on the normalized MRI exams by adding to each voxel an intensity value randomly selected from a distribution with mean 0 and standard deviation of 0.1. No other data augmentation was performed.

The 741 included exams were randomly divided into 600 training exams, 41 validation exams, and 100 test exams. During an initial hyperparameter tuning phase, the 600 training exams and 41 validation exams were used to optimize two training hyperparameters: (1) the learning rate step decay schedule and (2) the number of training epochs. The goal of this process was to help the neural network optimization process find a high-performing solution (mean whole-tumor Dice coefficient,^27^ described below). Consequently, the learning rate was kept high initially. This allowed the optimizer to take larger steps and search more of the parameter space. After this initial coarse search, the learning rate was reduced at regular intervals to encourage the optimizer to hone in on the most promising solutions.

We found that learning effectively stopped and network performance plateaued after 50 epochs and five learning rate reductions (by which time the learning rate was $3.125\times 10^{-5}$). These learning rate reductions were applied at epochs 20, 30, 35, 40, and 45, determined empirically at points where the training and validation accuracy had converged. Variations in the timing of the rate reductions within ±3 epochs of this schedule had little impact on network performance. The key insight gained was to keep between 3 and 10 epochs between rate reductions, after the initial coarse search phase. Extending the number of epochs beyond 50 likewise did little to improve network performance.

At epoch 50, we performed a stochastic gradient descent warm restart^28^ (SGDR). Briefly, this operation has been shown to improve the performance of deep neural nets, especially when the parameter space may include multiple distinct near-optimal minima. We suspected this may have been the case with our dataset due to its extensive variability. SGDR was accomplished by setting the learning rate back to 0.001 and continuing optimization for a further 24 epochs. During this period, the learning rate was halved at each of the following epochs: 59, 62, 65, 68, and 71. This learning rate schedule was determined empirically by observing when accuracy metrics had converged, at which point we lowered the learning rate for further refinement of the model parameters.

Training was conducted on Amazon Web Services (AWS, Seattle, Washington) using an Amazon Machine Instance (AMI) customized for DL by Nvidia Inc (Santa Clara, California). The AMI ran on an AWS p3.2xlarge instance equipped with an Nvidia Tesla V100 GPU, 8 Intel Xeon processors, and 64 GB of RAM. All training data were anonymized prior to being uploaded to Amazon Elastic Block Storage, where it was available to the p3 instance for processing.

Once the hyperparameter tuning phase was complete, training of an ensemble of five networks for brain tumor segmentation began. Each instance of the DeepMedic network was initialized with random weights and then trained from scratch. The training process described above was followed, except the validation exams were included in the training dataset. Thus, the number of training exams was increased to 641. No validation set was used during ensemble training. The 100 test exams remained sequestered during this process.

Training required an average of 28 h and 51 min per ensemble instance. A total of 144 h and 15 min of execution time were required to train the entire ensemble of five networks. Multiple AWS virtual machines were used in parallel to reduce the elapsed training time. Once trained, each instance in our ensemble required an average of 791 s to segment the brain tumors in all 100 test exams (7.91  s/exam). A total of 3953 s was required for all five ensemble instances to segment all 100 test exams (39.53  s/exam). These times include both data transfer and processing. In theory, five AWS virtual machines could be used in parallel, one per ensemble instance, to reduce the elapsed segmentation time per exam to ∼8  s.

Agreement between the technician and DL segmentations was evaluated using the Dice coefficient.^27^ This value varies between 0 and 1 and indicates the degree of overlap between the 3D lesion segmentations. A value of 0 indicates no overlap, while a value of 1 indicates perfect overlap. The Dice coefficient was determined for each of the 100 test cases.

### 2.4 Neuroradiologist Review

**Fig. 1:**
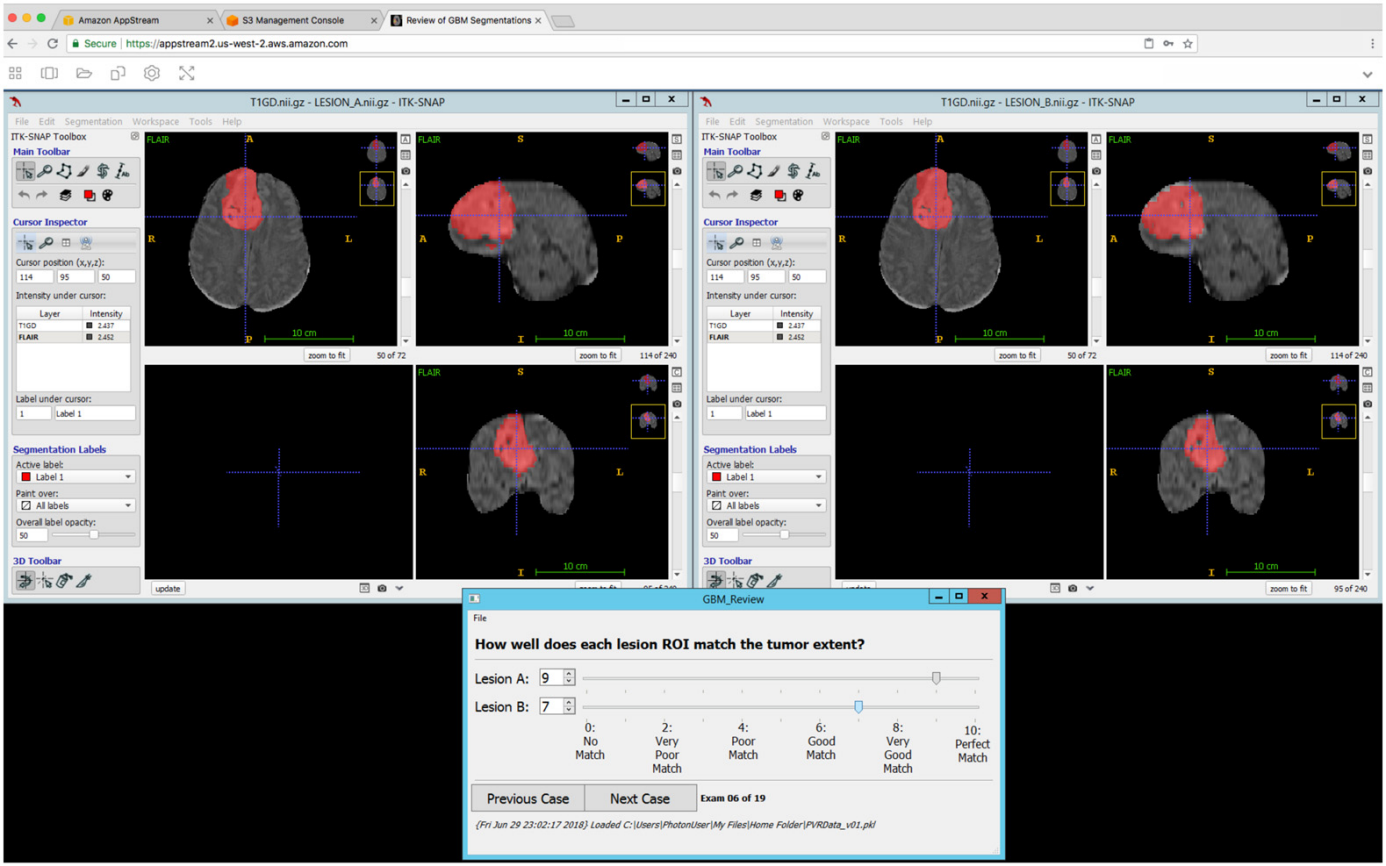
Our segmentation review software running on AWS AppStream 2.0. AppStream allows the developer to run Windows in a virtual machine on AWS and display the output to a remote instance of Google Chrome. Any application that can be installed in Windows can be installed in the virtual machine. We developed our own application in Python 3.6 and QT 5. The program launched two instances of insight segmentation and registration toolkit (ITK)-SNAP (windows top right and top left) to display an MRI exam from the test set along with the manual technician and automatic DL tumor segmentations (red overlays). The order of the display is randomized, and the viewer is blinded to the source of the segmentation. Lesion A is always displayed in the top-left window and lesion B in the top right. The viewer can zoom in and out and move the cursor (crosshairs) to any location in the MRI volume. The two ITK-SNAP instances are synchronized so that they show the same location at all times. The bottom window provides widgets (sliders) that allow the viewer to quickly and easily score the quality of each segmentation. The bottom window also provides widgets that allow the viewer to move forward (or backward) through the MR exams in their assigned group of exams.

A review of the 100 test cases was performed by 20 board-certified neuroradiologists [1 from Moffitt Cancer Center and 19 from Mayo Clinic, including Rochester, Minnesota (12); Phoenix, Arizona (6); and Jacksonville, Florida (1)]. The radiologists’ numbers of years of work experience, postcertification in neuroradiology, ranged from 1 to 23 years with a mean (±standard deviation) of 14.2 (±8) years. The radiologists were asked to compare the technician and DL segmentations by viewing them side-by-side, then scoring each on a scale of 0 through 10 (Fig. 1). The radiologists were instructed to assign scores based on how well each segmentation matched the tumor extent visible in the MRI exam. They were informed that a score of 0 indicated that the segmentation had no overlap with the MRI visible tumor, while a score of 10 indicated that the segmentation perfectly matched the MRI visible tumor. The slider widgets used for specifying scores allowed the radiologists to specify integer values between 0 and 10. The sliders were enumerated as follows: 0, no match; 2, very poor match; 4, poor match; 6, good match; 8, very good match; and 10, perfect match.

The order of the displayed exams was randomized, and the radiologists were blinded to the source of the segmentation. Due to the workload involved, each radiologist was asked to perform 20 side-by-side comparisons. Therefore, the 100 test exams were randomly split into five groups, each containing 20 of the test exams. Each radiologist was randomly assigned to one of the five groups. Thus, each group of 20 test exams was examined by four independent radiologists (20 radiologists divided by five groups). In total, 400 side-by-side comparisons and evaluations were performed (20 radiologists times 20 exams per radiologist).

The review was performed using a custom-developed program running on the AWS AppStream 2.0 application streaming service. AppStream supports programs that can execute on Microsoft Windows Server 2012 R2 (Microsoft Inc., Redmond, Washington). The Windows operating system runs on a virtual machine. User input to, and graphical output from, this virtual machine is streamed over a secure https connection to/from an instance of the Google Chrome web browser (Google Inc., Mountain View, California) running on a remote device. This service allows the developer to select from a range of virtual machines with varying hardware capabilities. We used the “stream.graphics-design.large” virtual machine instance in this experiment.

The 100 test exams and their associated manual technician and automatic DL segmentations were uploaded to 100 separate folders inside the AppStream virtual machine. Each exam folder contained four files: the T1c and FLAIR MRI volumes and the technician and DL segmentations. All files were stored in NIfTI format. The segmentations in each folder were assigned a generic name, either “A” or “B,” since this name was visible in the review application (below). The order of A and B was randomized between the technician and DL segmentations for each test exam.

The segmentation review application was written in Python 3.6 and used the QT v5 framework for its graphical interface (Fig. 1). The application displayed a window that allowed radiologists to step through the test exams in their assigned group, one at a time, forward or backward. The window also included widgets to allow the radiologists to easily enter and save segmentation scores.

The Python application launched two instances of ITK-SNAP^29^ to display each test exam and segmentations. Each ITK-SNAP instance loaded both the T1c and FLAIR volumes. The ITK-SNAP instance displaying segmentation A was positioned in the top left of the display, while the ITK-SNAP instance displaying segmentation B was positioned in the top right. The Python program invoked the Windows application “AutoHotKey”^30^ to arrange the ITK-SNAP and scoring windows on the display. When the radiologist chose to display the next (or previous) exam in their group, the Python program terminated the two ITK-SNAP processes and then repeated the process described above for the next (or previous) exam in the assigned group.

ITK-SNAP provided axial, sagittal, and coronal views of the MRI volumes and segmentations. The segmentations were displayed as translucent overlays on top of the MRI volumes. The radiologists could change this transparency, alter the intensity contrast settings for either displayed MRI volume, and position the cursor and view anywhere within either MRI volume. The two ITK-SNAP instances were “synced” so that cursor position and display remained the same in both instances at all times showing the exact same 2D MRI slices.

Radiologists could complete their reviews over multiple sessions—all program state and scoring information was preserved between sessions. After a radiologist completed his or her group of 20 reviews, a single binary, machine-readable file containing all of their scores was retrieved from their AppStream account for analysis.

## 3 Results

Our study included 741 exams from 729 unique patients. The 741 exams had the following sex distribution: 451 males, 262 females, and 28 unspecified sex. The mean (±standard deviation) age of the patients was 53.5 (±16) years (Table 1). The cohort included 525 MRI exams from eight North American institutions, 204 exams from three public domain datasets, and 12 exams from a consortium (Table 1). Included MRI exams ranged in date from 1990 to 2016, with a median acquisition year of 2006. The cohort contained 19 different tumor types (Table 2). The most common tumor type was glioblastoma (449 of 741 exams or 61%). About 145 exams (20%) had a tumor type that was not specified.

**Table 1:** Primary sources for the exams processed in this study. In total, eight North American academic cancer centers, three public domain datasets, and one consortium dataset contributed exams. “Study source” indicates the origin of the MRI exams. “N” indicates the number of exams contributed. “Age” is the mean age (±standard deviation) of the patients when the exam was obtained. “M/F (not specified)” indicates the number of male (M) and female (F) patients in the group. The number of patients whose sex was not specified is indicated in brackets. “Study dates” lists the range of years the exams were acquired, with the median year indicated in brackets. The last row provides summary values for the entire cohort.

	Study source	N	Age	M/F (not specified)	Study dates
1	Cancer centers (n=8)	525	53.1±15.9	338/187	2000 to 2016 (2008)
2	TCGA-GBM	101	58.4±14.4	63/38	1996 to 2008 (2001)
3	TCIA	85	45.6±15.6	33/24 (28)	1990 to 2005 (1994)
4	Ivy GAP^a^	18	56.7±13.4	7/11	1996 to 2000 (1997)
5	Radiation therapy oncology group	12	66.9±17.0	10/2	2009 to 2011 (2010)
	Overall	741	53.5±16.0	451/262 (28)	1990 to 2016 (2006)

a Ivy Glioblastoma Atlas Project.

**Table 2:** The different types of brain tumors and their frequencies, as reported in the patient cohort.

Tumor type			N	(%)
Glioblastomas			463	62.5
1	Glioblastoma multiforme	449	—	—
2	Glioblastoma multiforme with oligodendroglial component	7	—	—
3	Giant cell glioblastoma	4	—	—
4	Glioblastoma multiforme, small cell type	2	—	—
5	Glioblastoma multiforme with sarcomatous differentiation	1	—	—
Astrocytomas			77	10.4
6	Astrocytoma	38	—	—
7	Anaplastic astrocytoma	28	—	—
8	Diffuse astrocytoma	7	—	—
9	Infiltrating fibrillary astrocytoma	2	—	—
10	Gemistocytic astrocytoma	1	—	—
11	Pleomorphic xanthoastrocytoma	1	—	—
Oligodendrogliomas			37	5
12	Oligodendroglioma	27	—	—
13	Anaplastic oligodendroglioma	10	—	—
Mixed and other			19	2.5
14	Anaplastic oligoastrocytoma	9	—	—
15	Gliosarcoma	5	—	—
16	Oligoastrocytoma	2	—	—
17	Ganglioglioma	1	—	—
18	Diffuse pontine intrinsic glioma	1	—	—
19	Low-grade glioma	1	—	—
Not specified			145	19.6
Total			741	100

This dataset included 1482 3D MRI volumes (2 per study), 75,045 2D MR images (mean: 101 images per study or 50 2D images per 3D MRI volume), and 2337 technician-generated 3D tumor segmentations (mean: 3.2 segmentations per study or 1.6 segmentations per MRI volume).

**Fig. 2:**
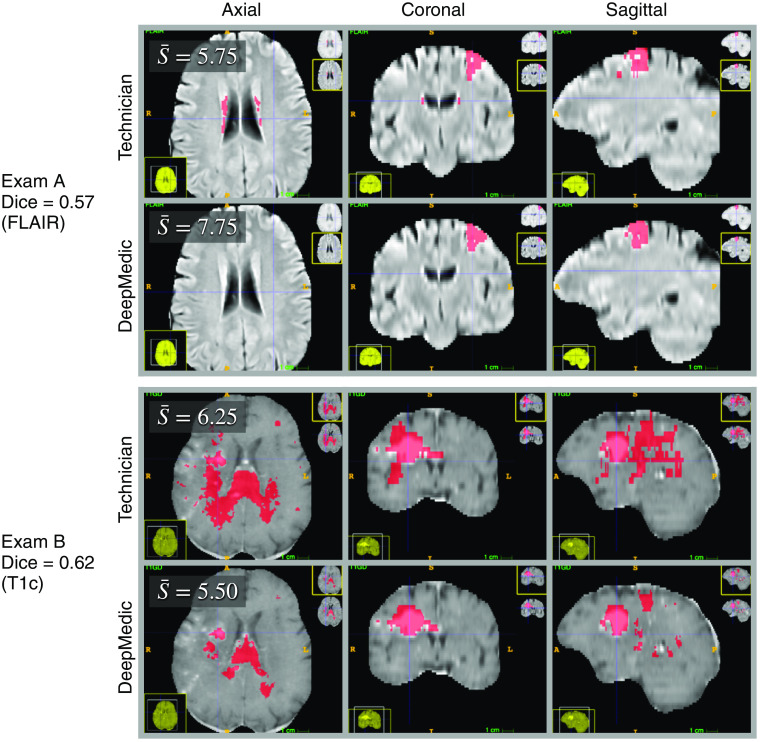
The two test exams with the lowest Dice coefficients (poorest agreement between the technician and DeepMedic defined tumor regions) among the 100 test exams. Tumor segmentations are indicated in red. The mean neuroradiologist score for each exam, $\overset{¯}{S}$, is displayed in the top-left corner of the axial view. Exam A (top two rows) had the lowest Dice coefficient among the 100 test exams. The disagreement between the two segmentation sources occurred primarily in the periventricular regions, where the technician labeled hyperintense regions as tumor, while DeepMedic did not. Periventricular hyperintensities are linked to small blood vessel disease and increased risk of stroke and dementia.^31^ Their prevalence increases with age in the general population. However, they typically are not associated with neoplasia. Exam B (bottom two rows) was tied with another exam (not shown) for the second lowest Dice coefficient. The disagreement in exam B was widespread. Both segmentations missed areas of enhancement in the T1c scan.

**Fig. 3:**
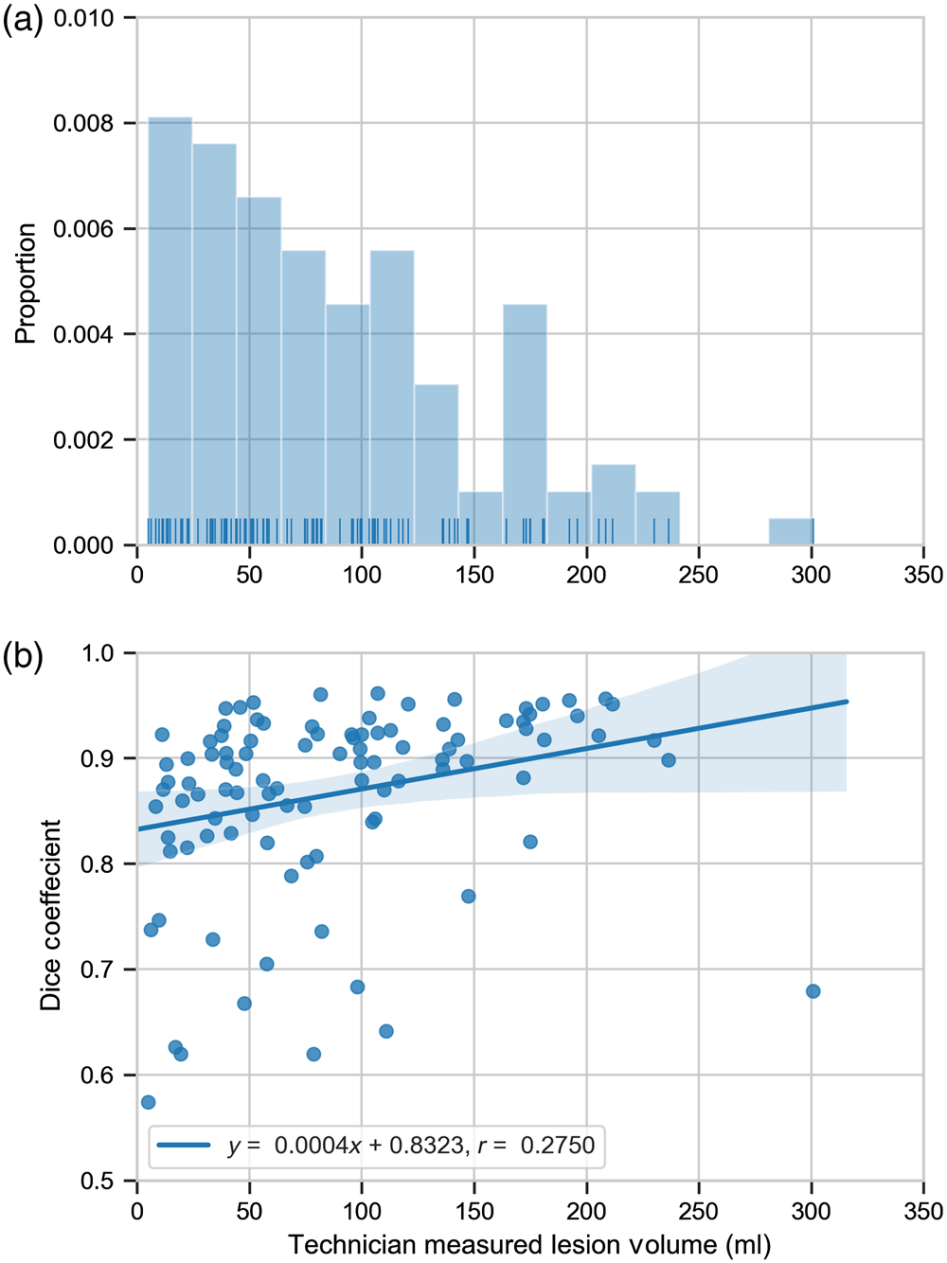
The distribution and Dice coefficients of the tumor volume measured by the technician. (a) The distribution of tumor volumes. These ranged from 5.07 to 300.84 ml with a mean (±standard deviation) of 88.98 (±62.68) ml. The median technician measured tumor volume was 78.20 ml. (b) Linear regression (blue line) between Dice coefficients and technician measured tumor volumes. This fit suggests a slight increase in Dice coefficient with increasing lesion volume (slope=0.0004). However, this relationship is weak (r=0.2750). The shaded blue region indicates the 95% confidence interval of the linear regression.

The whole-tumor mean and median Dice coefficient, recall, and precision values over the 100 test cases are given in Table 3. The two test exams with the lowest Dice coefficients are shown in Fig. 2. Figure 3(a) shows the distribution of technician measured lesion volumes. Figure 3(b) shows the relationship between Dice coefficients and technician measured lesion volumes. This figure suggests a slight increase in Dice coefficient with increasing lesion volume (slope=0.0004), although the relationship is weak (r=0.2750).

**Table 3:** The whole-tumor mean (±standard error), median Dice coefficient, recall, and precision over the 100 test cases. Values range from 0 to 1 in each case, with higher values indicating better performance.

	Dice	Recall	Precision
Mean	0.87 (±0.01)	0.87 (±0.01)	0.88 (±0.01)
Median	0.90	0.91	0.90

**Fig. 4:**
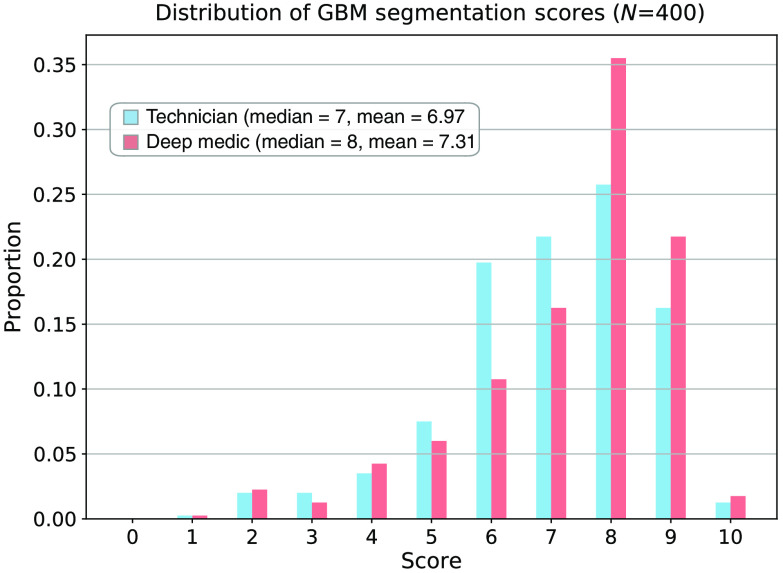
The distribution of scores for manual technician and automatic DL segmentations in the test exams. Twenty neuroradiologists each performed 20 blinded and randomized side-by-side comparisons of the technician and DL segmentations in the 100 test exams. Scores ranged from 0 (no overlap with the MRI visible tumor) to 10 (perfect match with the MRI visible tumor). The technician and DL segmentations had median scores of 7 and 8 and mean (±standard error) scores of 6.97±0.12 and 7.31±0.13, respectively. The magnitude difference in the mean scores was 0.34. This value was different from 0 with a two-sided p-value<0.00007. Additional details are provided in the text.

**Fig. 5:**
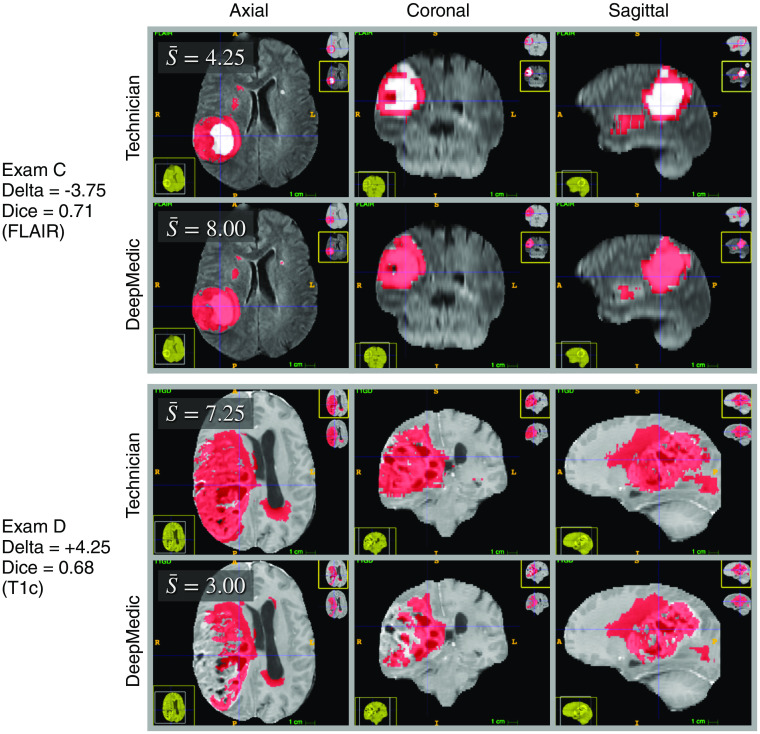
The two test exams with the largest differences (deltas) between the neuroradiologists’ mean scores for the technician and DeepMedic segmentations. Tumor segmentations are indicated in red. The mean neuroradiologist score for each exam, $\overset{¯}{S}$, is displayed in the top-left corner of the axial view. Delta is defined as ${\overset{¯}{S}}_{\text{Technician}}-{\overset{¯}{S}}_{\text{DeepMedic}}$. Exam C (top two rows) had the largest score difference in favor of the DeepMedic segmentation. The technician did not label the enhancing core of the tumor in exam C. Exam D (bottom two rows) had the largest score difference in favor of the technician segmentation. DeepMedic did not label extensive regions of enhancement in the T1c scan in exam D.

**Fig. 6:**
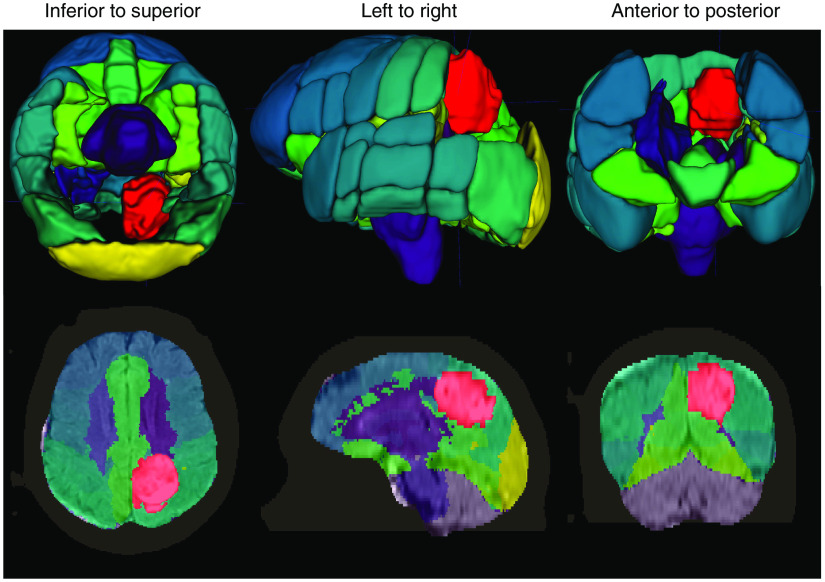
Example output from our DL system for automatic brain tumor segmentation. The system loads an MRI exam containing a T1-weighted postcontrast scan (T1c) and a FLAIR scan. Input from a wide range of MRI scanners and with varying scan parameters will work. We designed the system to perform the following steps automatically, without additional input: (1) enhance the MRI scans to remove artifacts; (2) identify the brain within the MRI scan (strip the skull), even in the presence of significant pathology or surgical interventions; (3) segment the brain tumor; and (4) coregister the Harvard-Oxford probabilistic atlas to the brain. The last step is used for visualization purposes and is optional. In this image, the tumor is red. Other colors indicate various atlas regions. The top and bottom rows show 3D and 2D views of the output data, respectively. Several atlas regions in the vicinity of the tumor have been made transparent in the 3D view to aid tumor visualization.

The neuroradiologist scores for the technician and DL segmentations had median values of 7 and 8 and mean (± standard error) values of 6.97±0.12 and 7.31±0.13, respectively (Fig. 4). The magnitude difference in the mean scores was 0.34. This value was different from 0 with a two-sided p-value<0.00007. The two test exams with the largest differences between the neuroradiologists’ mean scores for the technician and DL segmentations are shown in Fig. 5. Figure 6 shows an example output from our processing pipeline.

## 4 Discussion

Recently, several groups have reported results from DL systems for brain tumor segmentation (Table 4). The accuracy of these, and prior, systems has generally been assessed by measuring the overlap with manual segmentations. The most commonly reported metric is the Dice coefficient. There are limitations with this approach—manual labeling is challenging and subject to variability. Also, even well trained and experienced technicians occasionally make mistakes (see Figs. 2 and 5). Hence measures such as Dice may not accurately reflect real quality.

**Table 4:** The agreement between manual and DL tumor segmentation, expressed as the mean or median Dice coefficient over the test set for multiple neural nets. The Dice coefficients for the Heidelberg datasets are for contrast-enhancing tumor regions. Dice coefficients for all other entries are for whole-tumor segmentation. “MRI series” is the number of series required as input. “Val. Set Size” refers to the validation set size. The first three deep nets were the top-scoring solutions for the multimodal BraTS challenge from 2017. Networks 4 through 7 were the top-scoring solutions from BraTS 2018. The Heidelberg solution was trained using a fivefold cross-validation on 455 exams, i.e., the dataset was divided into five groups of 91 exams each. In each fold, four of these groups (364 exams) were used for training and one group (91 exams) was used for validation. The resulting five deep neural networks were then used as an ensemble to segment a separate sequence of 239 exams from the same institution. Then, the Heidelberg ensemble was used to segment 2034 exams acquired from 38 institutions as part of a clinical trial (EORTC). DeepMedic is our ensemble of five networks applied to 100 of our test studies. Additional details are provided in the text.

Neural network		Dataset	MRI series	Ensemble size	Training set size	Val. set size	Test set size	Test median Dice	Test mean Dice
1	EMMA^12^	BraTS 2017	4	21	285	46	146	N/A	0.88
2	Cascaded CNNs^11^	BraTS 2017	4	9	285	46	146	N/A	0.87
3	Brain Tumor U-Net^13^	BraTS 2017	4	15	285	46	146	N/A	0.86
4	NVDLMED^32^	BraTS 2018	4	10	285	66	191	0.92	0.88
5	MIC-DKFZ^33^	BraTS 2018	4	10	285	66	191	0.92	0.88
6	DeepSCAN^34^	BraTS 2018	4	12	285	66	191	0.92	0.89
7	DL_86_61^35^	BraTS 2018	4	7	285	66	191	0.92	0.88
8	Heidelberg^14^	Heidelberg EORTC	4	5	364	91	2273	0.89 to 0.91	N/A
9	DeepMedic	This study	2	5	641	0	100	0.90	0.87

Therefore, an important contribution of this work was to evaluate the quality of the DL segmentations via the first comprehensive and objective comparison of automated and human segmentation using a blinded controlled assessment study. On average, the neuroradiologists scored the automated DL segmentations higher (better) than the manual technician segmentations by 0.34 points on a 10-point scale. This difference had a p-value<0.00007.

Current top performing systems tend to have median and mean Dice coefficients near 0.92 and 0.88, respectively (Table 4). All of the experiments given in Table 4 made use of four MRI sequences, except ours, which used only two. Our experiment utilized a state-of-the-art brain tumor segmentation system. Consequently, we suspect that the additional information provided by four sequences may be responsible for the 1% to 2% improvement in mean Dice coefficient over our results. On the other hand, requiring only two input sequences should make our method more practical in clinical workflows.

Review of our 741 exams, after training and testing were complete, revealed that exam quality varied. The dataset includes exams with motion artifacts, aliasing artifacts, minimal attenuation of the fluid signal in some FLAIR sequences, occasional unconventional orientations of the head inside the MRI scanner, and variation in the MRI acquisition parameters. The diversity of our training data provides some assurance that our method will be translatable,^36^ at least for segmentation of pretreatment lesions. Future work will include training DeepMedic with exams from our database acquired throughout treatment and follow-up.

We did not evaluate the performance of our network using the BraTS challenge dataset. This is because both our dataset and the BraTS dataset contain a significant number of common MRI exams—those from The Cancer Imaging Archive (TCIA) and The Cancer Genome Atlas Glioblastoma Multiforme (GBM) data collections (TCGA-GBM).^37^ Differences in the naming conventions between the BraTS dataset and ours prevented us from determining correspondence between specific MRI exams in the two datasets. Thus, there was a high likelihood that studies used to train our network were present in the BraTS data. Using our trained network to segment tumors in the BraTS dataset would have produced biased results.

**Fig. 7:**
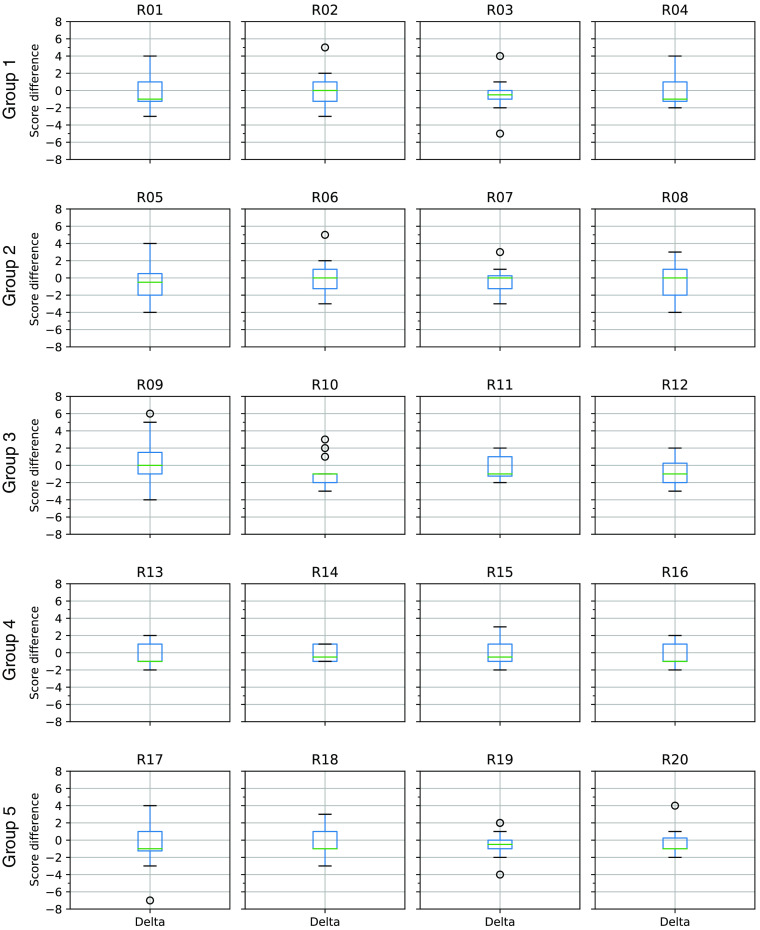
Boxplots showing the distribution of radiologists’ score differences by test group. The R numbers correspond to individual radiologists. For example, R01 refers to radiologist #1. Each row of plots corresponds to a specific group of 20 test exams. Thus, radiologists R01 through R04 all scored the same 20 exams in group 1. The score difference is defined as the radiologist score for the technician segmentation minus the radiologist score for the DL segmentation. Negative values indicate that the DL segmentation was assigned a higher (better) score than the technician segmentation. Each box shows the range of data values between the first and third quartiles. The horizontal line within each box indicates the median value. The whiskers indicate the range of values. Outliers are indicated by small circles beyond the whiskers. Variability between radiologists, both within and between groups, is evident as differing box sizes and whisker lengths.

We observed within- and between-radiologist scoring variability (Fig. 7). Consequently, the score differences between the technician and DL segmentations are likely to be even larger than suggested, if these differences are real.^38^ To determine the effects of scoring variability and the degree of agreement between all of the radiologists, we would need to perform a replication study in which multiple radiologists perform multiple repeated scores on a large number of segmentations.^8^^,^^39^^–^^41^ But ultimately, the challenge lies in the relative subjectivity intrinsic in human (even expert neuroradiologist) assessment.

Our blinded controlled assessment study indicates that our DL system produced higher-quality segmentations, on average, than the technicians who created the training labels. This observation appears to contradict the widely held belief that a model is only as good as the data used to train it. However, it should be noted that it is very difficult to determine the exact border of a cellularly diffuse and invasive tumor in the brain of a living patient. Consequently, our training labels likely include imperfections. The relationships between model accuracy, the number of training samples, and the effects of imperfect, or “noisy,” training labels have been studied extensively.^42^^–^^45^ These studies show that, in general, models achieve higher accuracy than the average accuracy of the training labels (provided that the labels have >50% accuracy). For example, Sheng et al.^42^ demonstrated an example in which 70% accurate labels were used to train a model that achieved 90% accuracy when applied to a sequestered test set with perfect labels. In the same publication, 80% accurate labels produced a model with near-perfect accuracy on the test set.

Finally, our study suggests that there may be new ways to use finite image labeling resources (limited by time and/or budget) to produce models with better overall performance. For example, rather than acquire a few high-quality segmentations, it may be better to acquire a larger number of lower-quality segmentations with additional repeated segmentations per lesion. We expect that the success of new strategies will depend upon many factors, including lesion complexity, the experience of the people performing the segmentations, the number of segmentations, and the methods used to extract information from repeated measurements. Additional studies are required to investigate the effects of these factors on model performance.

To our knowledge, this is the first time that this phenomenon has been demonstrated in a medical image segmentation task. There are several interesting ramifications. First, perfect or near-perfect training labels may not be required to produce high-performing segmentation systems. This could be important for any medical image segmentation task in which near-perfect labels are difficult, time-consuming, and/or costly to obtain. Second, the prior studies show that when labels are imperfect there are advantages to obtaining multiple labels for each training sample. Furthermore, there are several methods for combining information from repeated labeling to improve model performance.

## Code, Data, and Materials Availability

The data used in this study (741 MRI exams) are part of a larger collection curated by Dr. Kristin Swanson, Department of Neurosurgery, Mayo Clinic Arizona. These data contain protected health information and are therefore subject to HIPAA regulations. If requested, data may be made available for sharing to qualified parties as soon as is reasonably possible, so long as such a request does not invade subject privacy, or betray confidentiality. In particular, Mayo Clinic institutional policy is that MR images are identifiable (https://www.nejm.org/doi/full/10.1056/NEJMc1908881). While platforms may exist to completely deidentify images, the resources are not currently available to complete that process. Thus, sharing of images requires additional constraints to ensure protection of patient privacy. Typically, access to this type of data (inclusive of images) occurs through a collaboration and may require interested parties to obtain an affiliate appointment with Mayo Clinic and/or require a data-sharing agreement. Data that are shared will include standards and notations needed to interpret the data, following commonly accepted practices in the field. The custom software developed for this research project will be stored at Moffitt Cancer Center, with backup. This software will be published in archival journals, and made available to the scientific community upon request so long as such a request does not compromise intellectual property interests, interfere with publication, invade subject privacy, or betray confidentiality. Typically, software access will occur through a collaboration and may require a software sharing agreement.
